# Determinants of illness-specific social support and its relation to distress in long-term melanoma survivors

**DOI:** 10.1186/s12889-018-5401-1

**Published:** 2018-04-17

**Authors:** Sabine Fischbeck, Veronika Weyer-Elberich, Sylke R. Zeissig, Barbara H. Imruck, Maria Blettner, Harald Binder, Manfred E. Beutel

**Affiliations:** 1 Department of Psychosomatic Medicine and Psychotherapy, Medical Psychology and Medical Sociology, University Medical Center of the Johannes Gutenberg-University Mainz, Saarstr 21, D-55099 Mainz, Germany; 2 Institute for Medical Biostatistics, Epidemiology and Informatics (IMBEI), University Medical Center of the Johannes Gutenberg-University Mainz, Mainz, Germany; 3 Cancer Registry of Rhineland-Palatinate, Mainz, Germany; 4 Department of Psychosomatic Medicine and Psychotherapy, University Medical Center of the Johannes Gutenberg-University Mainz, Mainz, Germany; 5 Institute for Medical Biometry and Statistics, Faculty of Medicine and Medical Center - University of Freiburg, Freiburg im Breisgau, Germany

**Keywords:** Cancer, Oncology, Malignant melanoma, Long-term survivors, Social support, Distress

## Abstract

**Background:**

Social support is considered to be one of the most important resources for coping with cancer. However, social interactions may also be detrimental, e. g. disappointing or discouraging. The present study explored: 1. the extent of illness-specific positive aspects of social support and detrimental interactions in melanoma survivors, 2. their relationships to mental health characteristics (e. g. distress, quality of life, fatigue, coping processes, and dispositional optimism) and 3. Combinations of positive social support and detrimental interactions in relation to depression and anxiety.

**Methods:**

Based on the cancer registry of Rhineland-Palatinate, Germany, melanoma patients diagnosed at least 5 years before the survey were contacted by their physicians. *N* = 689 melanoma patients filled out the Illness-specific Social Support Scale ISSS (German version) and standardised instruments measuring potential psychosocial determinants of social support.

**Results:**

Using principal component analysis, the two factor structure of the ISSS could be reproduced with acceptable reliability; subscales were “Positive Support” (PS) and “Detrimental Interactions” (DI); Cronbach’s α = .95/.72. PS was rated higher than DI. Multivariable linear regressions identified different associations with psychosocial determinants. Survivors living in a partnership and those actively seeking out support had a higher probability of receiving PS, but not DI. PS and DI interacted regarding their association with distress: Survivors reporting high DI but low PS were the most depressed and anxious. High DI was partly buffered by PS. When DI was low, high or low PS made no difference regarding distress.

**Conclusion:**

Psycho-oncologic interventions should take into account both positive and negative aspects of support in order to promote coping with the disease.

## Background

Survivors of melanoma and their families face multiple illness-related practical and emotional demands, e. g. fears of recurrence and physical burdens [1]. Significant individuals such as the partner, family members, friends or physicians, nurses and other health professionals have the potential to provide instrumental, emotional or informational social support counteracting adverse effects of the disease [2–4]. Social support has been predictive for health related quality of life (HRQOL) and well-being [5], illness-coping and tumour-related anxiety [6–8]. A lack of social support has been associated with illness-related burden [9] and lower quality of life, particularly in individuals suffering from comorbid depression [10, 11] or distress [12].

Compared to breast cancer, patients suffering from melanoma have reported less social support; female patients sought more social support than male patients [3]. More positive social support has been reported when melanoma patients lived in a partnership [8]. Older patients received less support than younger ones and men compared to women [3]. Positive social support was positively correlated with cognitive and active coping [8, 13, 14], but only the patients’ emotional approach coping was associated with the correspondence between patients’ and partners’ reports of social support [6]. A significant, but low correlation was found between time elapsed since diagnosis and poor emotional support [8]. Stronger social support, fewer intrusive thoughts, and avoidance before treatment were associated with better adjustment one month after treatment, and social support deficiency was associated with higher depression, anxiety and lower quality of life [15]. Optimism elicited social support and reduced depression [16]. In later stages of disease progression, social support appeared to be enhanced [14, 17].

Research in this area has mostly inquired associations between the extent of social support, quality of life and distress. But there have been only a few studies to date which investigated the role of unsupportive interactions, termed detrimental interactions. However, as the metaphor of a “double edged sword” implies, significant individuals may also adversely affect the well-being of chronically ill patients, by being critical, unsupportive or neglectful when patients expects support and understanding [18]. Detrimental interactions enhanced depression and anxiety and reduced the dignity of cancer patients [19]. In accordance with the social-cognitive process model, the quality and extent of social support and coping processes interacted, influencing adjustment to cancer [15].

Indeed, relationships between coping and social support in melanoma patients may work both ways: Social support has been associated with coping processes [15, 20] and coping has been shown to predict social support [8, 17]. To our knowledge, combined effects of positive and detrimental support on distress have not been studied in long-term melanoma survivors. As a first step we wish to analyze which factors enhance or diminish social support and which promote detrimental interactions. Furthermore, we want to investigate, if specific patterns of support are associated with lower or higher distress. E.g., positive effects of social support could be reduced by a harmful effect of detrimental interactions. Both aspects might be essential in order to develop effective and targeted interventions for long-term survivors.

Based on the previous research findings, the aims of the present study were threefold:

1. to explore the extent of illness-related supportive and detrimental interactions in long-term melanoma survivors,
2. to identify illness-related and psychosocial determinants of both positive support and detrimental interactions,
3. to investigate combinations between positive social support and detrimental interactions in relation to depression and anxiety.

## Methods

### Study design and participants

All patients with a diagnosis of malignant melanoma (ICD-10: C43) who had been registered by their dermatologist at the Cancer Registry of Rhineland-Palatinate as part of routine documentation were eligible. Inclusion criteria were a) registration in the time period from 2000 to 2005, b) age of 14 years or older at diagnosis, and c) signed written informed consent for study participation. We excluded those patients who a) were not informed about their registration and b) did not have adequate German language comprehension or cognitive ability to participate. For legal reasons and in order to insure confidentiality, only the Cancer Registry was allowed to decode the name of the patient. Only the dermatologist who had originally registered the patient was eligible to contact the patient, who was then provided with a written informed consent form and the questionnaire. If more than one physician had reported the same patient to the registry, we decided to contact the hospital in-patient dermatological department first. The reporting physician received the letter for the potential study participant from the study center. The physician checked and updated the address of the former patient, informed the study center about possible reasons for non-participation of the patient (e.g. deceased, mentally disabled) and sent the signed letter back to the confidential office of the cancer registry. If the documents were not returned after six weeks, the physician received a reminder. If there was no response after another six weeks, the physician was contacted via phone a maximum of two times (first and second phone-in session). If the physician agreed to take part in the study, documents were sent to him/her again if necessary. If he/she refused or was unavailable after the 2nd phone-in session, no further contact was attempted. Study information, informed consent, questionnaires and the signed form letter of the physician were sent to the study participants by the study center. Patients who did not react to the letter within six weeks received a reminder letter and were contacted once more. Those who still did not respond were not contacted further. Data analysis was performed with coded data without reference to any personal identification. 72 out of 112 contacted dermatologists participated in the study (64.3%). Reasons for physician non-participation (*n* = 40) were “no interest in any kinds of studies or particularly in studies on QOL” (*n* = 23), retiring from their medical office (*n* = 5), “lack of time/too much bureaucracy” (*n* = 3) or “no personal contact with the patient anymore” (*n* = 1).

There was an average time delay of four months between the first contact with dermatologists and the first contact with patients. Of all contacted patients (*n* = 1320), *n* = 689 questionnaires were returned. Thus, 52.2% of the contacted patients took part, representing 32.6% of all registered patients. We were not able to contact 19.5% of the patients because the corresponding dermatologists did not participate in the study. There were no statistically significant differences between participants and non-participants (patients with a theoretical chance of being contacted via their treating dermatologist, who finally did not take part in the study; *n* = 1012) regarding tumor site, cancer stage, time since diagnosis and place of residence (urban/rural), but there were slight differences regarding the age distribution and sex in the univariate analysis: Non-participants were older (Median test *p* < .0001) and more often female (Chi2-test <.05) [21]. Sociodemographic and medical characteristics of the study sample (extracted from the data pool of the MELA study [22]) are listed in Table 1.

**Table 1:** Demographic and medical characteristics of the sample (*n* = 689)

	Number	Percent
Age (lowest 28, highest 93)		
≤ 39	52	7.6
40–49	111	16.1
50–59	137	19.9
60–69	133	19.3
70–79	184	26.7
≥ 80	72	10.5
Gender: male	335	48.6
Marital status		
Married	520	75.5
Single	49	7.1
Separated/divorced	49	7.1
Widowed	70	10.2
Partnership: yes	570	82.7
Educational level		
Primary school	333	48.3
Secondary school	196	28.4
High school	142	20.6
Other	17	2.5
Time since diagnosis (years)		
6	104	15.1
7	139	20.2
8	129	18.7
9	107	15.5
10	96	13.8
11	88	12.9
12	26	3.8
Social class		
Lower	237	34.4
Middle	234	34.0
Higher	218	31.6
UICC stage at diagnosis^a^		
1	365	53.0
2	34	4.9
3	7	1.0
Unknown	283	41.1

^a^UICC-stage: till 2003 (year of diagnosis) according to TNM 5. ed., Springer publisher 1997, from 2004 (year of diagnosis) TNM 6. ed., Springer publisher 2002; classification according to TNM 6 in A and B were subsumed to the particular stage; missing data: marital status (1), partnership (23), educational level (1), melanoma surgery (9)

### Ethics statement

The protocol was approved by the Ethics Committee of the Statutory Physician Board of the State of Rhineland-Palatinate (Reference number 837.161.11, 7703).

### Measures

We assessed social support with the Illness-specific Social Support Scale (ISSS) [18], German version [23], because the ISSS assesses both positive and negative aspects. In the present sample principal component analysis reproduced the two scales “positive support” (PS, 15 items) and “detrimental interaction” (DI, 9 items). Items were scored on a 5-point Likert scale from 0 (“never”) to 4 (“always”). Internal consistency in the MeLa data set was Cronbach’s α =.94 (positive support) and α = .72 (detrimental interactions). In accordance with Revenson et al. [18], positive and problematic interactions were unrelated. Construct validity was supported by the pattern of correlations with selected standardised questionnaires (see Table 2). High and low degrees of positive social interactions, and detrimental interactions were defined by median split in order to analyse effects of combinations of positive or detrimental interactions.

**Table 2:** Illness-Related Social Support Scale (662 < *n* < 676)

Items of the scales	M	SD	r_it_	a_1_	a_2_	h^2^
Among the people close to you, there is someone who						
Positive support (Cronbach’s α = .95)						
1. Listens to you.^a^	3.25	0.97	.74	.79		.64
2. Does small favours for you^a^, e.g. goes shopping or looks after the children.	2.53	1.40	.60	.64		.42
4. Helps out in a crisis, even if they have to go out of their way.^a^	2.81	1.26	.72	.75		.57
5. Makes you feel you have something positive to contribute to others.^a^	2.69	1.16	.69	.74		.54
7. Gives you information or advice if you want it.^a^	2.56	1.27	.70	.75		.56
8. Is available for you whenever you need it.	3.38	0.98	.75	.80		.64
11. Boosts your spirits.^a^	2.77	1.27	.80	.84		.70
14. Cheers or comforts you.^a^	2.81	1.24	.82	.85		.72
15. Gives you positive feedback about how you are coping.^a^	2.27	1.40	.66	.71		.51
18. Gives up some of their time and energy to help with something that needs to be done.^a^	2.49	1.33	.70	.74		.56
20. Tells you that you are a worthwhile person.^a^	2.74	1.16	.73	.77		.60
22. Helps you to explore alternatives.^a^	2.09	1.30	.66	.70		.50
23. Gives you the feeling that you are important to him/her.	3.19	0.98	.76	.80		.66
25. Shares the upset with you.^a^	2.61	1.34	.69	.74		.55
26. Talks about important decisions with you.^a^	3.15	1.13	.74	.78		.61
Scale values	2.60	0.87				
Detrimental interactions (Cronbach’s α = .72)						
3. Seems to feel awkward when you talk about the disease with him/her.	0.93	1.18	.41		.58	.34
6. Worries too much or is pessimistic towards your disease.	0.95	1.06	.33		.48	.26
9. Becomes annoyed when you don’t accept their advice.^a^	1.33	1.17	.37		.54	.32
10. Plays down the impact of your disease.	0.65	.98	.44		.60	.39
12. Gives you the feeling that you are incapable of taking care of yourself.	0.48	.92	.39		.53	.28
17. Tries to change the way you are coping in a way you don’t like.^a^	0.51	1.00	.34		.49	.25
19. Gives you information or makes suggestions, that you find unhelpful or upsetting.^a^	0.79	1.05	.37		.53	.28
21. Expects more from you than you can handle.	0.88	.99	.45		.61	.44
24. Finds it hard to understand the way you feel.^a^	1.17	1.06	.47		.63	.45
Scale values	0.85	0.56				

*M* mean, *SD* standard deviation, *r*_*it*_ power, α Cronbach‘s Alpha, *N* sample size; ^a^original ISSS-item

*ISSS* Illness-specific Social Support Scale. Based on a 5-point Likert Scale from 0 = “never” to 4 = “always”

The numeration equates to the order of the items in the original questionnaire

Principal component analysis, 49.01% of total variance explained, item and scale analysis, a1/2 = factor loadings, h^2^ = communality

Depression was measured using the reliable and validated German version of the Patient Health Questionnaire Depression Module (PHQ-9) [24]. Caseness of depression was defined as a score of PHQ-9 ≥ 10, indicating moderate to severe depressive symptoms for the past two weeks. Unless otherwise noted Cronbach’s of this and the following measures in the MeLa data set has been reported by Fischbeck et al. [25].

The Generalized Anxiety Disorder Questionnaire (GAD-7) of the PHQ was used to assess generalised anxiety disorder.

We used the Quality of Life Core Questionnaire EORTC-QLQ-C30 [26], which measures cancer-related quality of life reliably and with good validity using five functional scales (physical, role, cognitive, emotional, social; the three symptom scales: pain, fatigue, nausea/vomiting, a global health and quality of life scale, and six single items assessing additional symptoms commonly reported by patients (e.g. appetite loss, sleep disturbance) as well as the perceived financial impact of disease.

Fatigue was assessed using the Multidimensional Fatigue Inventory (MFI), an internationally validated, multidimensional self-administered instrument [27] with the subscales general, physical and mental fatigue, reduced motivation, and reduced activity.

The Brief Cope (BC), a short version of the COPE questionnaire [28, 29], has proven useful in health-related research. As more than half of the scales had a Cronbach α< .70, we performed an exploratory factor analysis leading to three interpretable coping scales (accounting for 38% of total variance): The dimensions “seeking external support” (α = .75), “denial/self-blaming” (α = .74), and “positive reappraisal/active coping” (α = .76) were in good accordance with other Brief-Cope-related factor-analytic results [30, 31].

Psychosocial care needs were assessed by the 9 item Hornheide Questionnaire, short form (HQ-S) [31], including physical and mental well-being, tumour-related anxiety, tension, self-esteem, social support, support of the physician, and informational needs of skin cancer patients (cancer-related distress). A cut-off score ≥ 16 (or an item-based cut-off score) indicated need for psychosocial support [32–34].

We used the Life Orientation Scale (LOT-R) [35] in a German form [36] for the assessment optimism and pessimism as personality dimensions. It is the most commonly used instrument for measuring these constructs in psychological research, it has acceptable psychometric properties.

Sociodemographic data were obtained using a standardised self-reporting questionnaire. Illness-related information (UICC stage, tumour site, time since diagnosis) was extracted from the Rhineland-Palatinate Cancer Registry.

### Statistical analysis

Descriptive statistics were used to evaluate the sociodemographic patient and illness-related data. Social class was determined using a common index [37]. In order to determine the effects of the socio-demographic data (gender, age, marital status, social class), social and medical variables (partnership, medical issues: UICC status, complications, time since diagnosis), comorbidity (depression PHQ-9, anxiety GAD-7), and psychosocial variables (scales of the MFI, Brief COPE, EORTC (Symptoms, Global Health, Functioning), Life-orientation (LOT-R) and burden (HQ-S) on positive support and detrimental interactions, a linear regression model was fitted. We present adjusted beta weights, the corresponding 95% confidence intervals and *p*-values. Because of missing values in the potential predictor variables, multiple imputation was performed [38] with k = 10 imputed data sets via the SAS procedure MI. For two randomly chosen single imputation data sets, we selected a set of covariates with a linear regression model using forward and backward selection with a selection level of 5%. For each single imputation data set, we then performed linear regression models for the previously selected covariates and combined the results via the SAS procedure MIANALYZE.

In order to compare groups of patients with high and low support, we used the median splits of the two subscales (support low/detrimental interactions high, support high/detrimental interactions high, support high/detrimental interactions low, support low/detrimental interactions low). We performed Kruskall-Wallis and Mann-Whitney-U-Tests to compare distress (depression, anxiety) in these four subgroups. Because the tests were regarded as exploratory, no adjustments for multiple testing were done. *P*-values are given for descriptive reasons only and should be interpreted with caution. Statistical analysis was performed with the Statistical Package for the Social Sciences (SPSS, Chicago, IL, USA, version 21.0) and SAS 9.2 for Windows 9.2 TS Level 1 M0 (SAS Institute Inc. Cary, NC, USA).

## Results

### Sample

Our sample included *n* = 689 malignant melanoma survivors (354 women and 335 men; see Table 1). Nearly two thirds of the participants were 50 years of age or older. Most were diagnosed at stage UICC 1 (no spread or distant sites, 53%), and the melanoma was located in the extremities (47.7%). At the time of the study, 69.5% had lived with their illness for 6 to 9 years, 31.5% for 10 years or more. Most were married (75.5%), 82.7% had a partner, and 72% lived in rural communities. From the *n* = 689 participants, *n* = 13 had more than 10% missing data in the ISSS and were excluded from the data set.

### Perceived social support

The melanoma survivors reported receiving moderate to high levels of social support, as can be seen in Table 2. The ISSS mean scores of the total sample were M = 2.60 (SD = 0.87) for positive support and M = 0.85 (SD = 0.56) for detrimental interactions. Items representing the availability of people (items 8, 1) scored the highest. Among the comparatively frequent detrimental interactions was someone who becomes annoyed when the patient does not accept advice (item 9) or not feeling understood (item 24).

### Determinants of positive support and detrimental interactions

We assumed that overall positive support and detrimental interactions would be associated with demographic (sex, age, marital status, educational level, social class, partnership), disease and treatment variables (time since diagnosis, tumour stage, complications), disease-related burden (illness-related quality of life, distress, depression, anxiety, fatigue), and personality factors (coping, optimism/pessimism).

Results of the multivariable linear regression analysis using the complete case analysis (after multiple imputations) are shown in Table 3. Living in a partnership, seeking out social support (BC), and optimism (LOT-R) were positively, while being separated/divorced, having a self-blaming/defensive coping style (BC), pessimism (LOT-R), and general and physical fatigue (both MFI scales) were negatively associated with positive support (all, *p* < 0.05). Furthermore, melanoma survivors with a self-blaming/defensive coping style (BC), pessimism (LOT-R), general fatigue, and reduced motivation (both MFI scales) reported more detrimental interactions (all *p* < 0.05). Comparable variance was explained for positive support (range R^^2^ = 0.3236–0.3529) and detrimental interactions (range R^^2^ = 0.2722–0.3221).

**Table 3:** Multivariable linear regression on positive support and detrimental interactions (ISSS), n = 689

	*ß* ^a^	95% confidence limits		*p*-value
Positive support (PS)		lower	upper	
Marital status: separated/divorced	- 0.42	- 0.7258	- 0.0774	0.0160
Partnership	0.30	0.0524	0.5428	0.0174
Optimism (LOT-R)	0.11	0.1131	0.1990	0.0151
Pessimism (LOT-R)	- 0.19	- 0.2677	- 0.0213	< .0001
Searching external support (BC)	0.69	0.5663	0.8030	< .0001
Self-blaming/defense (BC)	- 0.23	- 0.4330	- 0.0229	0.0293
General fatigue (MFI)	- 0.04	- 0.0670	- 0.0123	0.0045
Physical fatigue (MFI)	- 0.05	- 0.0769	- 0.0172	0.0020
Detrimental interactions (DI)				
Pessimism (LOT-R)	0.07	0.0136	0.1175	0.0135
Self-blaming/defensive (BC)	0.33	0.1759	0.4757	< .0001
General fatigue (MFI)	- 0.02	- 0.0390	- 0.0023	0.0286
Reduced motivation (MFI)	0.03	0.0091	0.0477	0.0041

^a^ Multiple Imputation for a set of covariates selected by forward and backward selection (level of selection 5%) in single imputation data

PS: R˄^2^ = 0.3236–0.3529, Not in the equation: age, gender, educational level, married or single, social class, time since diagnosis, tumour stage, complications, illness-related quality of life, cancer-related distress, depression, anxiety, fatigue (activity, motivation, mental), positive reappraisal/active coping

DI: R˄^2^ = 0.3236–0.3529), Not in the equation: age, gender, educational level, marital status, partnership, social class, time since diagnosis, tumour stage, complications, illness-related quality of life, cancer-related distress, depression, anxiety, fatigue (physical, activity, mental), coping, dispositional optimism

### Distress and social support

Table 4 determines the associations of different extents of positive support and detrimental interactions with depression and anxiety. Based on median split, the highest depression scores could be found when detrimental interactions were high and support was low (PHQ-9 M = 6.45, SD = 5.0), followed by high detrimental interactions and high positive support (PHQ-9 = 2.91, SD = 3.03). When detrimental interactions were low, positive social support made no difference regarding depression. In terms of anxiety scores, the same patterns of results were found. The highest proportion of distressed study participants was observed under the support pattern detrimental interactions high and positive support low (over cut-off score: 24% depression, 24% anxiety). The lowest proportion we registered under the pattern positive support and detrimental interactions low (depression: 2%, anxiety 3%).

**Table 4:** Associations of combinations (groups split by median) of positive support (PS) and detrimental interactions (DI) with depression (PHQ-9) and anxiety (GAD), means and SD; proportion of depression and anxiety over cut-off score (oc) related to support pattern

		PS low	oc	PS high	oc
DI low	depression	2.63 (2.68), *n* = 134	2%	2.91 (3.03), *n* = 146	4%
	anxiety	2.10 (2.57), *n* = 140	3%	2.72 (2.57), *n* = 145	6%
DI high	depression	6.45 (5.00), n = 134	24%	3.97 (4.39), *n* = 138	9%
	anxiety	5.54 (4.70), n = 145	24%	3.88 (4.19), *n* = 145	15%
Comparison of subgroups:					
Depression			Anxiety		
(Kruskall Wallis U-Test Chi^2^ = 67.186, df 3; p < .001): PS low-DI high vs. PS high-DI high p < .001, PS low-DI high vs. PS high-DI low *p* < .001, PS low-DI high vs. PS low-DI low *p* < .001, PS high-DI high vs. PS high-DI low *p* < .05, PS high-DI high vs. PS low-DI low p < .05, PS high-DI low vs. PS low-DI low n. s.			(Kruskall Wallis U-Test Chi^2^ = 69. 868 (df 3), p < .001): PS low-DI high vs. PS high-DI high p < .001, PS low-DI high vs. PS high-DI low p < .001, PS low-DI high vs. PS low-DI low p < .001, PS high-DI high vs. PS high-DI low p < .001, PS high-DI high vs. PS low-DI low p < .001, PS high-DI low vs. PS low-DI low n. s.		

## Discussion

Research in melanoma patient has repeatedly found associations between social support, HRQOL and psychological co-morbidity. However, studies including long-term survivors have not been conducted to date. Which factors are associated with the perception of getting social support has remained unanswered. Non-supportive transactions from members of the family or friends, which could diminish the effect of supportive transactions have found little attention. We therefore examined the contributions of sociodemographic, medical, distress, coping, quality of life and personality variables to two dimensions of social interactions with significant persons, positive support and detrimental (problematic) interactions. Overall, long-term survivors reported considerably higher scores for positive social support compared to detrimental interactions. Multiple linear regression analyses identified distinct associations with psychosocial determinants. Survivors living in a partnership and those actively seeking out support had a higher probability of receiving positive support, but reported less detrimental interactions. Both aspects of support interacted regarding their association with distress: Survivors confronted with high detrimental interactions, but low positive support, were the most depressed and anxious. When detrimental interactions were low, high or low social support made no difference regarding depression and anxiety.

In accordance with other studies [23, 39], living in a partnership was associated with positive support experiences and being divorced or separated was associated with lower positive support, but there were no differences related to detrimental interactions. Interestingly, being married did not predict either dimension. The quality of a partnership seemed to have supportive effects rather than the family status itself. This might be connected to the availability of support at home; problems dating a new partner are well known in cancer survivors [40]. Regarding the association of the personality variable optimism with positive support, an optimistic survivor has better preconditions to activate the support he or she needs. Optimists usually approach challenges with confidence and persistence, but pessimists are more often doubtful and reluctant [41]. A self-blaming and defensive coping style appeared to be a barrier towards getting positive support. This style, which mostly consisted of inadequate stress management behaviour (self-blaming, using alcohol, giving up solving problems etc.) probably inhibited searching social support. Coping efficacy has been known to mediate the relationship between spouses’ unsupportive behaviour and cancer patients’ psychological distress [42]. An inclusion of interventions that seek to strengthen the positive emotional support and coping assistance provided by spouses may be particularly beneficial for patients with poor coping efficacy [43].

As expected, searching external support as a kind of active coping was related to positive support. This is consistent with other melanoma studies [8, 13, 14] and supports the importance of the patients’ actively demanding social support.

In accordance with our hypotheses and with other study results [44], general fatigue and physical fatigue were associated with both, supportive and non-supportive interactions. Being exhausted may have reduced the opportunities for supportive interactions. On the other hand, reduced motivation is associated with reports of detrimental interactions, possibly resulting from inactivity on behalf of melanoma survivors.

Contrary to expectations, in multivariate analysis, gender, age, being married, social class, UICC grade, complications, or time since diagnosis were not associated with positive support. The same was true for quality of life, depression, anxiety or distress, although each was individually correlated with positive support. In multiple regression analysis, these variables may have been suppressed by personality factors (coping, optimism) and fatigue.

Consistent with expectations, a high level of detrimental interaction contributed to higher levels of distress, namely depressive and anxiety symptoms. Our results indicate that positive support cannot fully buffer the detrimental effect of problematic interactions, but can reduce it slightly. Despite an overall low level, the noxious effects of detrimental interaction on distress appeared to outweigh positive support. Similar to Revenson et al. [18], our findings supported the hypothesis, that personal relationships can be both a potential source of distress and a source of support for melanoma survivors, and their relative balance determines their association with depression and anxiety. Social support also encourages internal resources, whereas negative interactions may lead to negative attitudes toward the self or one’s dignity [19]. This is consistent with the interaction of positive support and detrimental interactions with regard to distress. Not surprisingly, the highest distress (depression, anxiety) was found when positive support was low and detrimental interactions were high. However, there was less, but still considerable, distress when detrimental interactions were high and positive support was high, indicating that positive social support did not completely buffer disappointment. When detrimental interactions were low, there were no differences between low and high social support groups related to distress. More social support may not necessarily lead to a greater reduction of depression and anxiety. An overprotection of partner could even be associated with more distress and less feelings of control by cancer patients [45].

Advantages and limitations of our study and areas for future research should be mentioned:

The fact that the sample was registry-based insured its representativeness, but the registry was not allowed to contact patients directly. This required cooperation not only from the former patients but also from their physicians many years after cancer diagnosis and treatment. While within the range of comparable studies (e. g. [46, 47]), the response rate of 52.2% in our study restricted the validity of our results. Missing data in the cancer registry concerning initial tumour stage led to information gaps. As there were few survivors from progressive disease 5 years after registration, our results are not generalizable for this patient group. Since non-participants were older and more often female, and these groups may be more often depressed than younger or male persons, representativeness of our results may have been reduced. A strength lay in the use of a broad range of standardised self-report scales, including the measurement of ‘illness-related social support’. Because the current study was cross-sectional, it was not possible to evaluate causal relationships between the explanatory (personal data, coping, optimism, quality of life) and criterion variables (positive support, detrimental interactions).

Even though this study focused on long-term survivors, no information was available about the patients’ social support levels at the time of diagnosis and during the melanoma treatment phase. If, how, why and when social support processes have changed is not known. Furthermore we could not identify, if the sources of support or detrimental interaction are the same or if there were within- or cross-domain buffering [48] effects on depression and anxiety. Longitudinal (intervention-) studies could clarify the development of support gaps, and if professional help could help to enhance support and decrease unsupportive interactions.

Our study alerts caretakers, psychooncologists and researchers not only to inquire and intervene regarding positive support, but also to take possible negative effects of social interactions into account. Importantly, positive support does not fully compensate for detrimental disappointing interactions which may be more relevant for a successful adaptation to cancer than positive aspects.

## Conclusions

Unlike most previous studies, we differentiated positive support and detrimental interactions as two independent dimensions of illness-related support. Multivariable regression revealed different determinants of the two facets of social support. Receiving social support was substantially associated with actively searching it. Although the diagnosis of melanoma was more than five years ago in our sample, some survivors may not be capable or unwilling to inform others about their need of support. Medical staff should be sensible to hidden needs. Self-blaming and defensive coping was associated with a higher risk for encountering detrimental interactions. Detrimental interactions were associated with distress and were only partially compensated by positive social support, a fact that has important implications for psychooncologic care. It is not enough to just encourage positive social support: it is at least as important to address disappointing and rejecting interactions in psychosocial interventions. Thus, psychooncologic interventions of long-term survivors may enhance the reception of helpful support and train assertiveness for empowering patients to articulate their needs or object to non-supportive interactions.

## Abbreviations

BC: Brief Cope
DI: Detrimental Interactions (DI)
EORTC-QLQ-C30: EORTC Quality of Life Core Questionnaire
GAD-7: Generalized Anxiety Disorder Questionnaire
HF-S: Hornheide Questionnaire, short form
ICD-10: International Classification of Diseases, Version 10
ISSS: Illness-specific Social Support Scale ISSS
LOT-R: Life Orientation Scale
M: Mean value
MFI: Multidimensional Fatigue Inventory
PHQ-9: Patient Health Questionnaire, Depression Module
PS: Positive Support
SD: Standard deviation
UICC: Union internationale contre le cancer
